# Perceptual-Cognitive Expertise in Elite Volleyball Players

**DOI:** 10.3389/fpsyg.2013.00036

**Published:** 2013-03-07

**Authors:** Heloisa Alves, Michelle W. Voss, Walter R. Boot, Andrea Deslandes, Victor Cossich, Jose Inacio Salles, Arthur F. Kramer

**Affiliations:** ^1^Lifelong Brain and Cognition Laboratory, Department of Psychology, Beckman Institute, University of Illinois at Urbana-ChampaignUrbana, IL, USA; ^2^Department of Psychology, Aging Mind and Brain Initiative, University of IowaIowa City, IA, USA; ^3^Department of Psychology, Florida State UniversityTallahasse, FL, USA; ^4^Exercise Neuroscience Laboratory (LaNEx/PPGEF), Universidade Gama FilhoRio de Janeiro, Brazil; ^5^Neuromuscular Research Laboratory, National Institute of Traumatology and OrthopaedicsRio de Janeiro, Brazil

**Keywords:** cognition, expertise, sport

## Abstract

The goal of the current study was to investigate the relationship between sport expertise and perceptual and cognitive skills, as measured by the component skills approach. We hypothesized that athletes would outperform non-athlete controls in a number of perceptual and cognitive domains and that sport expertise would minimize gender differences. A total of 154 individuals (87 professional volleyball players and 67 non-athlete controls) participated in the study. Participants performed a cognitive battery, which included tests of executive control, memory, and visuo-spatial attention. Athletes showed superior performance speed on three tasks (two executive control tasks and one visuo-spatial attentional processing task). In a subset of tasks, gender effects were observed mainly in the control group, supporting the notion that athletic experience can reduce traditional gender effects. The expertise effects obtained substantiate the view that laboratory tests of cognition may indeed enlighten the sport-cognition relationship.

## Introduction

For nearly three decades researchers have sought to better understand the psychological factors that discriminate expert athletes from less skilled athletes and non-athletes (Abernethy, 1987; Starkes and Alard, 1993; Starkes and Ericsson, 2003). It has been widely demonstrated that fitness training improves cognitive functioning and changes structural and functional aspects of the brain (Dustman et al., 1990; Colcombe and Kramer, 2003; Etnier et al., 2006; Kramer and Erickson, 2007). Similarly, cognitive training can improve basic attention and perceptual skills, and higher-level cognition (Ball et al., 2002; Erickson et al., 2007; Basak et al., 2008). However, although research focusing on perceptual-cognitive skill in sport is abundant, it is still unclear whether years of extensive sport training is associated with superior performance on tests of basic perceptual and cognitive processes.

It has been demonstrated that housing animals in enriched environments positively influences brain organization as well as learning and memory (Park et al., 1992; Van Praag et al., 1999). Similarly, Fabel et al. (2009) demonstrated that physical activity and exposure to an enriched environment, although potentially acting through different mechanisms, are additive in their effect on adult hippocampal neurogenesis in mice. Along the same line, it may be reasonable to suggest that a professional sport environment may represent a kind of enriched environment for humans since it entails physical and mental challenges. In other words, superior cognitive and perceptual performance may be observed in elite athletes, due to the combined effects of physical training and cognitive stimulation provided by the sport setting.

Traditionally, perceptual-cognitive expertise in sport has been studied through two theoretical approaches: the expert performance approach and the component skills approach. The expert performance approach studies the athlete in a sport-specific context (Starkes and Ericsson, 2003; Mann et al., 2007), allowing experts to directly transfer skills from the field to the laboratory (near transfer). Overall, studies employing this approach have demonstrated that experts, when compared to non-experts, show more elaborate task knowledge, make more use of available information, encode, and retrieve relevant information more efficiently, visually detect and locate objects, and patterns in the visual field faster and more accurately, use situational probability information better, and make more rapid and appropriate decisions (Singer and Janelle, 1999; Williams et al., 1999; Starkes and Ericsson, 2003; Mann et al., 2007). In contrast, the component skills approach assesses the relationship between basic (i.e., not sport-specific) cognitive skills and sport expertise (Nougier et al., 1991; Starkes and Ericsson, 2003). The component skills approach has been criticized for not capturing the complexities of the environment that generates superior expert performance (Ericsson, 2003). However, it can determine whether athletes differ from non-athletes in more general perceptual and cognitive processes. In other words, it is able to capture skills that transfer to contexts outside of sport (far transfer). Although some studies do not support this view (Memmert et al., 2009), a recent meta-analysis by Voss et al. (2009) showed that high-performing athletes consistently outperformed non-experts in tests of a subset of cognitive abilities (processing speed and visual attention, although not attention cuing), as measured by the component skills approach.

Transfer of acquired skills is an important aspect of learning. One hypothesis is that transfer will occur if the trained and transfer tasks involve overlapping cognitive process and brain networks (Dahlin et al., 2008). This hypothesis may therefore provide an explanation for transfer from cognitive skills developed during sport training and similar processes outside of the domain of sport. A variety of cognitive and perceptual tests have been used to assess differences in cognitive skills between experts and non-experts. However, such research diversity has hindered the ability to compare effects across studies. Other factors have also limited the quantitative detail necessary to determine differences between experts and non-experts, such as the small sample sizes and the employment of a very limited number of cognitive tests per study. Lastly, most of the existing sport expertise studies have not included elite athletes in their analyses (mostly college athletes and amateur athletes). The present study addresses these issues thereby attempting to overcome some of the limitations of the previous studies described above. The primary goal of the study was to investigate the relationship between sport expertise and perceptual and cognitive skills, as measured by the component skills approach. To extend the types of tasks that have been used to examine athlete/non-athlete differences, the study employed a relatively broad cognitive battery that included cognitive tasks not previously used in the sport expertise literature. In addition, the study included a substantial number of professional athletes (including Olympic-level athletes), participants of both genders, and different age groups.

Our hypotheses were twofold. The primary hypothesis was that athletes would outperform non-athlete controls on the perceptual and cognitive tasks of the assessment battery (expertise effect). Specifically, we hypothesized that athletes would show: faster processing speed, higher accuracy rates, enhanced memory capacity, enhanced attentional breadth, greater selective attention, greater inhibitory control, and greater mental flexibility (i.e., ability to multi-task). The second hypothesis was that sport expertise would minimize gender differences. In the general population, a common finding is that females usually perform worse than males in reaction time (RT) measures (Seidel and Joschko, 1991; Ballard, 1996). However, other studies have shown that women consistently perform better than men on measures of verbal fluency and perceptual-motor speed (Kimura, 1983; Halpern, 2000; Weiss et al., 2003). A recent study demonstrated that video game training can virtually eliminate gender differences in spatial attention (Feng et al., 2007). Similarly, the results of the few studies that examined gender differences among athletes suggest that the finding of a female inferiority (or superiority) effect in the average population does not seem to generalize to female athletes (Lum et al., 2002). Along this vein, we predicted that gender differences, if they occurred, would only be observed in the control group, not in the athlete group.

## Materials and Methods

### Participants

A total of 154 individuals participated in the study. The athletes belonged to two distinct categories, according to age and years of training: adult and junior. Thirty adult players (21 men and 9 women) and 57 junior players (24 men and 33 women) were included in the sample. Twenty-seven non-athlete adult controls (18 men and 9 women) and 40 non-athlete young controls (18 men and 22 women) were also included in the sample. Table 1 presents the demographic information for the sample.

**Table 1:** **Sample demographics**.

Group	*N*	Age	Education	Total training
Adult male athletes	21	24.85 (4.40)	11.76 (0.94)	11.61 (4.75)
Adult female athletes	9	20.55 (1.23)	11.22 (1.09)	9.66 (1.5)
Junior male athletes	24	17.58 (0.92)	9.95 (0.88)	5.25 (2.43)
Junior female athletes	33	16.27 (1.06)	9.48 (1.14)	5.43 (1.94)
Adult male controls	18	23.33 (3.04)	14.61 (2.43)	–
Adult female controls	9	21.55 (1.50)	13.88 (1.38)	–
Junior male controls	18	17.33 (1.13)	10.33 (0.59)	–
Junior female controls	22	16.45 (1.53)	9.72 (0.88)	–

*Means and standard deviations (in parentheses) in years are given*.

All athletes were recruited at the Center for the Development of Volleyball (CDV – Saquarema), in Rio de Janeiro, Brazil. Control subjects were selected by word of mouth and through advertisements posted in classrooms in different universities and schools in the city of Rio de Janeiro. All participants completed a questionnaire prior to the beginning of the testing session, where they were asked to rate their health on a scale of 1 (poor) to 5 (excellent). Athletes were asked to specify the total number of years of volleyball training they had and the number of training hours they received every week. Although fitness level was not assessed in the control group, most adult participants were not involved in any kind of physical activity at the time of the intervention. Young control participants, on the other hand, participated in physical activities in school. All participants reported no major medical or psychological conditions, were not taking medication that would influence performance on the experimental tasks, and reported normal color vision and normal or corrected-to-normal vision acuity. Participants signed an informed consent form approved by the Institutional Review Board of the National Institute of Traumatology and Orthopaedics (INTO). Athletes and controls under 18 years of age had their consent forms signed by one of the parents, prior to the testing session.

### Procedures

The cognitive testing was conducted in a 2-h session. After completing the questionnaire, participants performed a cognitive battery, which included a number of computer-based tasks that were completed in the following fixed order: task switching, Useful Field of View (UFOV), Visual Short-Term Memory (VSTM), Stopping, Flanker, and Change Detection (see below for a description of each). Each task took 5–15 min to complete. During the testing session participants sat approximately 50 cm from the monitor.

#### Apparatus

Three Pentium 4 PCs, attached to 15′′ monitors, were used. The tasks were programmed with E-prime software (Psychology Software Tools, www.pstnet.com).

#### Cognitive battery

The tasks in the cognitive battery fell into three general categories: (a) executive control tasks (higher-level cognition), (b) memory tasks, and (c) visuo-spatial attentional processing tasks. All tasks are described below.

#### Executive control tasks (higher-level cognition)

##### Task switching

Task switching tests the ability to keep two tasks in mind at once and rapidly switch between tasks. Participants were asked to judge whether a number was odd or even, or whether it was high or low (i.e., larger or smaller than five). The color of the screen indicated which task participants had to perform on each trial. Randomly, the numbers 1, 2, 3, 4, 6, 7, 8, and 9 were presented, one at a time, for 1500 ms at the center of the screen on a pink or blue background. If the digit was on a blue background, participants had to respond as quickly as possible whether the number was low (“Z” key) or high (“/” key). If the digit was presented on a pink background, participants had to respond as quickly as possible whether the digit was odd (“Z” key) or even (“/” key). First, participants completed two practice single task blocks of 40 trials each (one block of odd/even and one block of high/low), followed by the respective single task blocks (also 40 trials each). Then participants completed a practice dual task block of 64 trials in which they switched from one task to another every five trials. Finally, participants completed a dual task block of 160 trials. During this block, it was randomly determined on each trial whether a trial required participants to respond high/low or odd/even.

An important measure of performance is task-switch cost during the dual task block: the difference in performance for trials when the preceding trial involved the same task (non-switch trial) and those when the preceding trial was of the other task (switch trial). Switch costs were calculated by subtracting the response time (RT) for non-switch trials from the response time for switch trials. Task-switch cost is an index of an aspect of executive control. A smaller switch cost indicates a greater ability to switch between two different tasks. This task is similar to that of Kramer et al. (1999) and Pashler (2000). Switch cost was also calculated for the accuracy variable, where the accuracy for the switch trials was subtracted from accuracy for the non-switch trials. In addition, RT and accuracy were analyzed for the different trial types (single task trials, non-switch, and switch trials).

##### Stopping

The stopping task measures inhibition of a motor response. Participants were asked to respond to a Z (left index finger) or a/(right index finger) as quickly as possible, as soon as it appeared on the screen. On 25% of trials, a tone occurred shortly after the appearance of the Z or/and participants were asked to inhibit their response when they heard this tone (stop trials). On the other 75% of trials, no tone occurred and participants were required to respond as quickly as possible by pressing Z or/(go trials). For stop trials, the tone was initially set to play 250 ms after the appearance of the letter. If participants successfully inhibited their response when the tone occurred, the delay between the letter and the tone was increased by 50 ms, making it harder for participants to inhibit their response the next time the tone occurred. If participants were unsuccessful in inhibiting their response, the delay between the letter and the tone was decreased, making it easier for participants to inhibit their response. The delay between the letter and tone was adjusted in this manner after each stop trial to find the delay at which participants were as likely to make a response as to withhold a response. A “stop reaction time,” a measure of inhibitory control, was calculated by subtracting the average delay between the letter and the tone from the average reaction time on go trials (Logan et al., 1997). RT and accuracy for the go trials and stop probability were also calculated. Participants completed 240 trials overall.

#### Memory

##### Visual short-term memory

On each trial, participants viewed four objects for 250 ms (the memory array). After 250 ms, the objects disappeared for 900 ms; then, one object was presented on the screen (the test object) and the task was to indicate whether the test object was one of the originally presented objects or not. Subjects were instructed to press Z if the object was present and/if it was not present in the original display. Two blocks of trials assessed memory for features. In the *color block*, four-color patches were presented. At test, a color patch was presented that was either the same as one of the color patches in the memory array (50% of trials) or different (50% of trials). In the *shape block*, four line drawings of different shapes (e.g., cross, heart, triangles, etc.) were presented in the memory array, and then the test object was either one of the objects in the test array or a different shape. Finally, in the *conjunction block*, four shapes in different colors were presented. Critically, on some trials, the test object had the same shape as one of the objects in the memory array and the same color as a different object in the memory array. Thus, the binding of features in memory was crucial to respond correctly. For each of the three conditions, participants completed 4 practice trials and 68 test trials. Accuracy is the primary measure of performance in this task.

#### Visuo-spatial attentional processing

##### Useful field of view

The UFOV task measures the breadth of visual attention. The ability to extract information from the periphery of vision is crucial to a number of important tasks, especially in sports. In the UFOV task, participants were asked to localize a target (a triangle within a circle 1.8° in diameter) briefly presented among square distractors (1.8° × 1.8°). Stimuli were arranged in eight radial spokes. Targets were presented with equal probability on each spoke at eccentricities of 4.5°, 9°, and 13.5° from fixation (Ball et al., 1988). A mask (100 ms) followed each search display. After search display and subsequent mask presentation, participants were asked to use the computer mouse to click on the spoke the target appeared on. There were five blocks of increasing difficulty levels, which were related to the duration of target presentation. First, participants performed three practice blocks, at three distinct difficulty levels: 75 ms (24 trials), 50 ms (24 trials), and 25 ms (48 trials). Then, participants performed two test blocks of 48 trials each, at a fixed difficulty level of 10 ms. Performance was measured by the accuracy of localizing the target (i.e., the average of the two test blocks at each eccentricity separately). When the target is presented further in the periphery, accuracy is typically poor. Interestingly, UFOV appears to be amenable with training (Roenker et al., 2003; Edwards et al., 2009).

##### Flanker

The flanker task measures selective attention: the ability to respond to relevant information while ignoring irrelevant or conflicting information. A set of five arrow-head shapes were presented on each trial (e.g., >>>>>). Participants were instructed to pay attention to the central arrow and ignore the flanking arrows. If the central arrow pointed to the left, participants were asked to press Z and if it pointed to the right, they were asked to press /. Two different trials were presented to participants: compatible (e.g., >>>>>) and incompatible trials (e.g., >><>>). Participants completed a practice block of 20 trials and a test block of 100 trials (conditions were equally distributed in both blocks, i.e., 50% compatible trials and 50% incompatible trials). Previous studies have found that subjects respond more slowly (and sometimes less accurately) when the flanking distractors are incompatible with the central target (e.g., >><>>). This is presumably due to an inability to restrict attention to only the task-relevant information. Response slowing on the incompatible trials has been shown to reduce with fitness training (Colcombe et al., 2004). The measures of this task are RT, accuracy, and flanker interference. Flanker interference was calculated by subtracting response time for compatible trials from response time for incompatible trials. Interference for accuracy was calculated by subtracting accuracy for incompatible from accuracy for compatible trials.

##### Change detection

The change detection task measures visual attention and memory. In this task, subjects searched for a difference between two different versions of a realistic scene. The displays consisted of images of driving scenes from the perspective of an automobile driver. Each trial began with an image presented on the screen for 240 ms, followed by a gray screen presented for 120 ms, and then the same image with one object in the scene changed for 240 ms. This sequence was repeated for 30 s, or until participants made a response. Participants were asked to find the change. Changes included color changes (e.g., cars changing color), location changes (e.g., pedestrians stepping into the road), and additions/deletions (e.g., signs appearing and disappearing). Upon finding the change, participants pressed C on the keyboard and then clicked with the mouse on the area of the image where the change had occurred. Participants performed 1 practice trial and 59 test trials. Every trial contained one change. The measures of performance in this task are mean RT for correct trials and accuracy.

## Results

The primary goal of the study was to investigate whether volleyball athletes and non-athlete controls differed in perceptual and cognitive abilities. Mean RT and accuracy data were entered into two multivariate analyses of covariance (MANCOVAs) with group, age, and gender as fixed factors, to determine the effects of sport expertise on different aspects of cognition. Although the two adult groups were matched for age, adult control participants had, on average, 3 years more of formal education than adult athletes (young controls and young athletes were matched for both age and education). Therefore, education was included as a covariate in the MANCOVAs. Outliers (i.e., participants with scores outside the range of 2.5 standard deviations from the mean of their particular group) were removed from the analyses. Analyses were conducted using SPSS (Version 11.5). Effect sizes, as measured by partial eta-squared ($\eta_{p}^{2}$), were computed. For each task, the measures that best represent the cognitive constructs relevant to the present investigation were selected for the purpose of the MANCOVAs.

In the RT MANCOVA, although education (Wilks’ λ = 0.98, *F* < 1) and gender [Wilks’ λ = 0.95, *F*(5,117) = 1.16, *p* = 0.33, $\eta_{p}^{2}=0.05$] were not significant, group [Wilks’ λ = 0.88, *F*(5,117) = 3.24, *p* = 0.01, $\eta_{p}^{2}=0.12$] and age [Wilks’ λ = 0.89, *F*(5,117) = 2.91, *p* = 0.02, $\eta_{p}^{2}=0.11$] were statistically significant. In the Accuracy MANCOVA, group (Wilks’ λ = 0.96, *F* < 1), age (Wilks’ λ = 0.99, *F* < 1), gender (Wilks’ λ = 0.97, *F* < 1), and education (Wilks’ λ = 0.95, *F* < 1) were not statistically significant.

Since the effect of Group was only significant in the RT MANCOVA, the analyses were followed by repeated measures and univariate analyses of variance (ANOVAs) on each task separately for all RT measures. Two tasks were not included in these subsequent analyses because their only measure of performance was accuracy, namely, the UFOV and VSTM tasks. Since education did not reach statistical significance in the MANCOVAs, it was not included as a covariate in the ANOVAs. Of interest were significant main effects of Group (i.e., expertise effect) and Group × Gender, Group × Age, and Group × Gender × Age interactions, which are reported below. Other significant results were not included here, since differences in cognition between the athlete and non-athlete groups were the main focus of the study. The results of each task are described separately. Mean and Standard Error (SE) are plotted for each variable analyzed. Practice blocks were not included in any of the analyses.

### Executive control

#### Task switching

**Figure 1:**
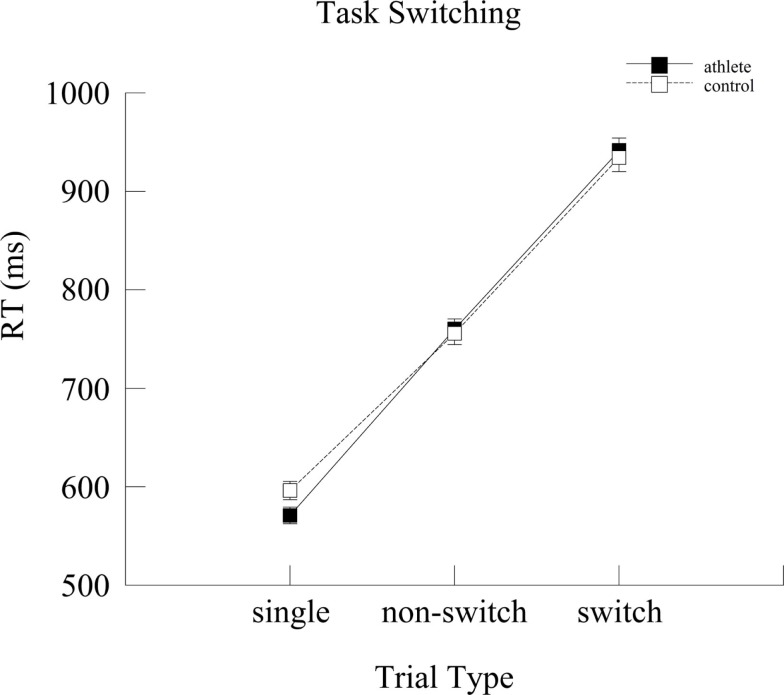
**Mean reaction time (ms) for the two groups as a function of trial type**. Error bars represent ±1 standard error.

A repeated measures ANOVA was run for the RT measure with Group (athlete and control), Age (adult and junior), and Gender (male and female) as between-subjects factors and Trial Type (single task trials, non-switch, and switch trials) as the within-subjects factor. A Group × Trial Type interaction was observed [*F*(2,276) = 2.95, *p* = 0.05, $\eta_{p}^{2}=0.02$], as illustrated in Figure 1. *Post hoc* tests showed that athlete group (570.88 ± 8.42 ms) was faster than the control group (596.18 ± 9.30 ms) exclusively on the single task trials (*p* = 0.05). To assess whether this difference was due to a speed-accuracy tradeoff, accuracy data on single trials were analyzed in a univariate ANOVA. However, no significant difference was observed between the groups (*p* = 0.33). Accuracy data for all speed-accuracy tradeoff analyses are reported in Table 2.

**Table 2:** **Mean accuracy values of the speed-accuracy tradeoff analyses**.

Task	Analysis/condition	Athlete group	Control group
Task switching	Single trials	0.92 (0.011)	0.93 (0.012)
Stopping	Go trials	0.98 (0.004)	0.98 (0.004)
Flanker	Females	0.97 (0.008)	0.97 (0.008)
Change detection	Group effect females	0.72 (0.013), 0.71 (0.019)	0.71 (0.013), 0.70 (0.021)

*Standard errors are given in parenthesis*.

### Stop and go responses

Separate univariate ANOVAs were employed to analyze Go RT, Stop RT, and Stop probability. As in the previous analysis, Group, Age, and Gender were included as between-subjects factors.

For Go RT (i.e., the response time when no tone occurred), the ANOVA yielded an effect of Group [*F*(1,139) = 16.14, *p* < 0.001, $\eta_{p}^{2}=0.10$], indicating that the control group (656.66 ± 16.45 ms) was faster than the athlete group (746.48 ± 15.13 ms). To test if this difference was due to a speed-accuracy tradeoff, Go accuracy data were analyzed in a univariate ANOVA. Controls and athletes did not differ (*p* = 0.29), rejecting this possibility.

**Figure 2:**
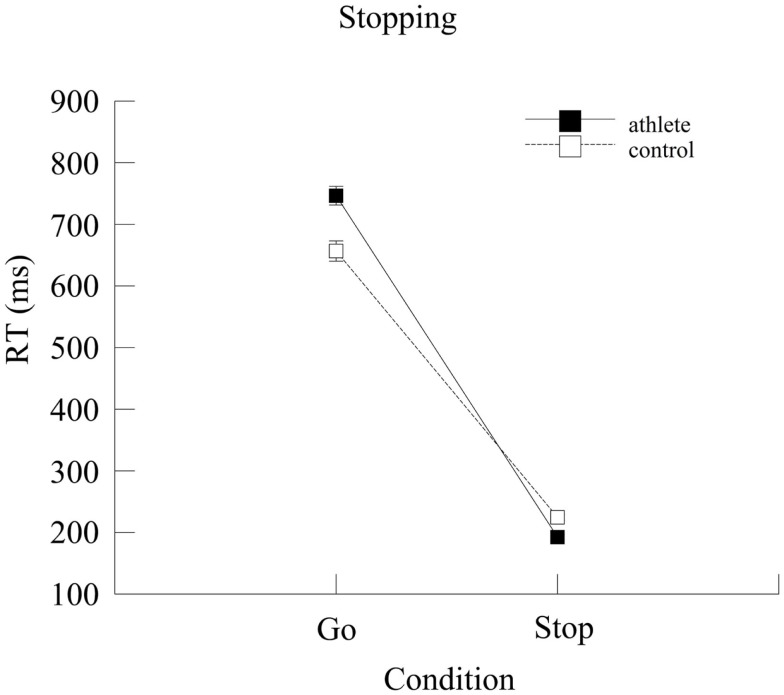
**Mean reaction time (ms) for the two groups on the Go and Stop conditions**. Error bars represent ±1 standard error.

For stop RT (i.e., the time taken to inhibit the response when the tone occurred), an effect of Group was also obtained [*F*(1,139) = 22.47, *p* < 0.001, $\eta_{p}^{2}=0.14$, but with the reversed pattern compared to the Go RT: athletes were faster to stop than controls (192.43 ± 4.59 and 224.58 ± 4.99 ms, respectively). Both results are depicted in Figure 2.

**Figure 3:**
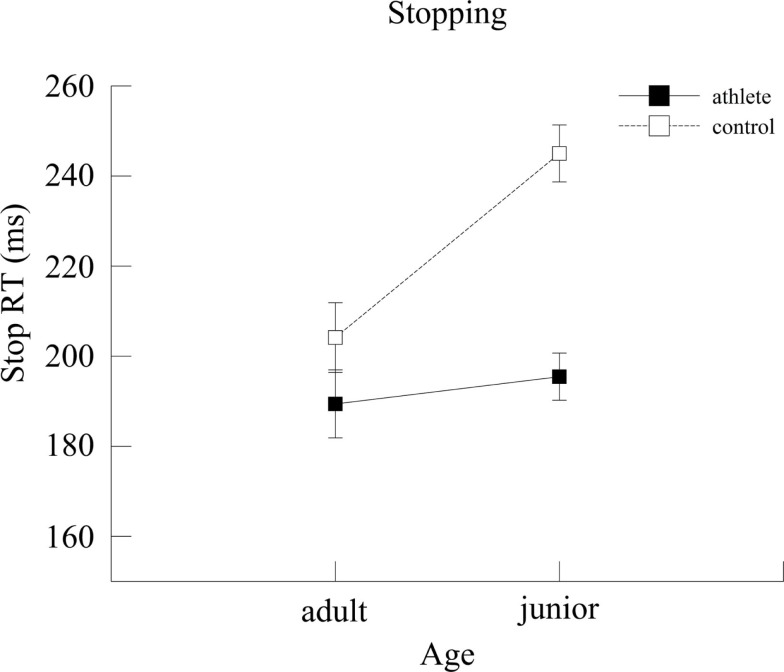
**Stop reaction time (ms) for the two groups as a function of age**. Error bars represent ±1 standard error.

A significant Group × Age interaction was observed [*F*(1,139) = 6.58, *p* = 0.01, $\eta_{p}^{2}=0.05$] for Stop RT (Figure 3). *Post hoc* analyses showed that there was no significant age difference in the athlete group (*p* = 0.52), while in the control group adult participants (204.15 ± 7.72 ms) were significantly faster to stop (*p* < 0.001) than junior participants (245.01 ± 6.31 ms). The analyses also showed that junior athletes (195.45 ± 5.24 ms) were significantly faster to stop (*p* < 0.001) than junior controls (245.01 ± 6.31 ms) but adult athletes and adult controls did not differ (*p* = 0.17).

**Figure 4:**
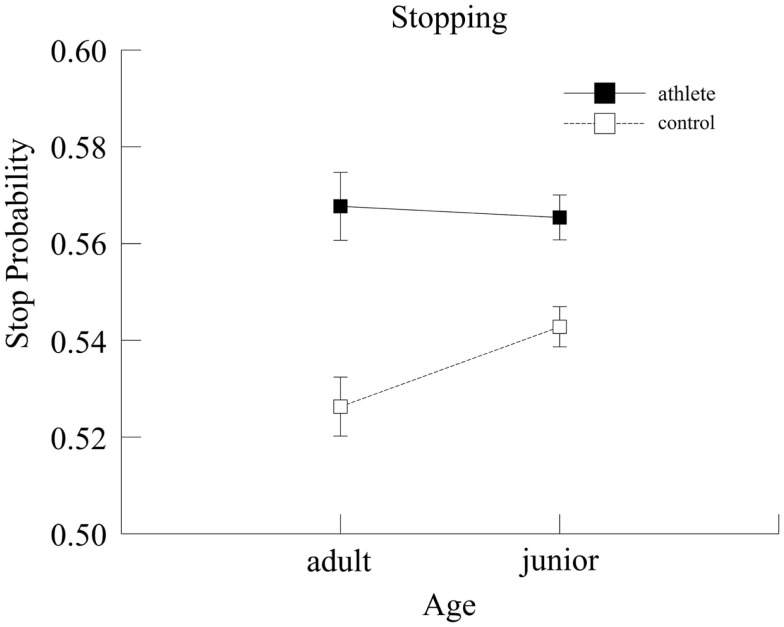
**Stop probability for the two groups as a function of age**. Error bars represent ±1 standard error.

For stop probability (i.e., the likelihood of stopping a prepotent response), a main effect of Group [*F*(1,139) = 31.39, *p* < 0.001, $\eta_{p}^{2}=0.18$] and a marginal Group × Age interaction [*F*(1,139) = 3.68, *p* = 0.06, $\eta_{p}^{2}=0.03$] were observed, as illustrated in Figure 4. The analysis indicated a higher probability of stopping in the athlete group. *Post hoc* analyses showed that there was no significant age difference between adult and junior athletes (*p* = 0.78) but junior controls (0.54 ± 0.01 ms) had a higher probability of stopping (*p* = 0.02) than adult controls (0.53 ± 0.01 ms). In addition, the analyses revealed that junior athletes (0.56 ± 0.01 ms) had a significantly higher probability of stopping (*p* < 0.001) than junior controls (0.54 ± 0.01 ms) and adult athletes had a higher probability of stopping (*p* < 0.001) than adult controls (0.57 ± 0.007 and 0.53 ± 0.01 ms, respectively).

### Visuo-spatial attentional processing

#### Flanker task

RT data were entered in a repeated measures ANOVA with Group, Age, and Gender as between-subjects factors and Condition (compatible and incompatible) as the within-subjects factor.

**Figure 5:**
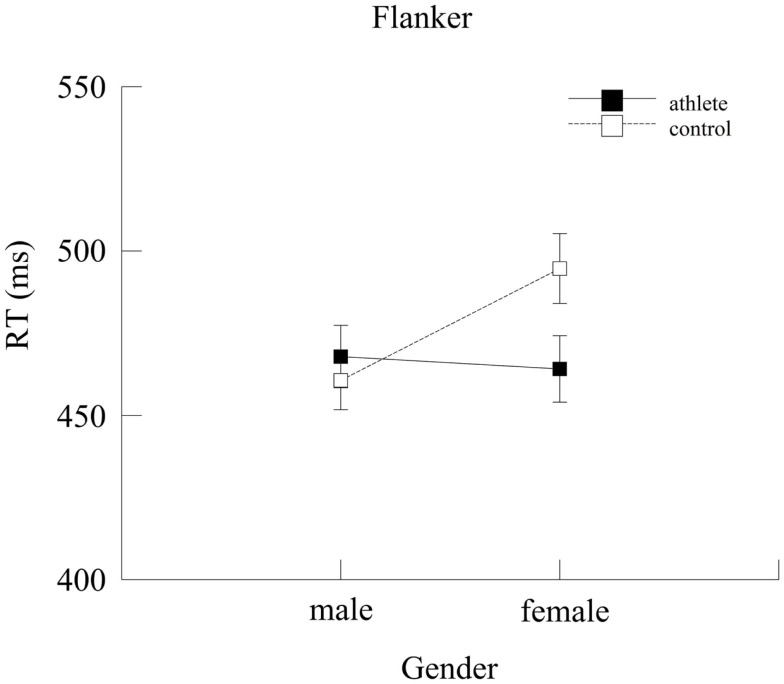
**Mean reaction time (ms) for the two groups as a function of gender**. Error bars represent ±1 standard error.

There was a marginal Group × Gender interaction [*F*(1,136) =3.70, *p* = 0.06, $\eta_{p}^{2}=0.03$], as shown in Figure 5. *Post hoc* tests revealed that there was no significant gender difference between male and female athletes (*p* = 0.98). On the other hand, male controls (460.64 ± 8.94 ms) were significantly faster (*p* = 0.01) than female controls (494.66 ± 10.61 ms). The analyses also indicated that female athletes (464.11 ± 10.12 ms) were faster (*p* = 0.02) than female controls (494.66 ± 10.61 ms). Again, to test if this difference was due to a speed-accuracy tradeoff, accuracy data were analyzed through an Independent Samples *t* test. The results indicated that female athletes were just as accurate as female controls (*p* = 0.97). Male athletes and male controls did not differ (*p* = 0.57). Main effects and other interactions were not statistically significant (*p*s > 0.05).

#### Change detection

**Figure 6:**
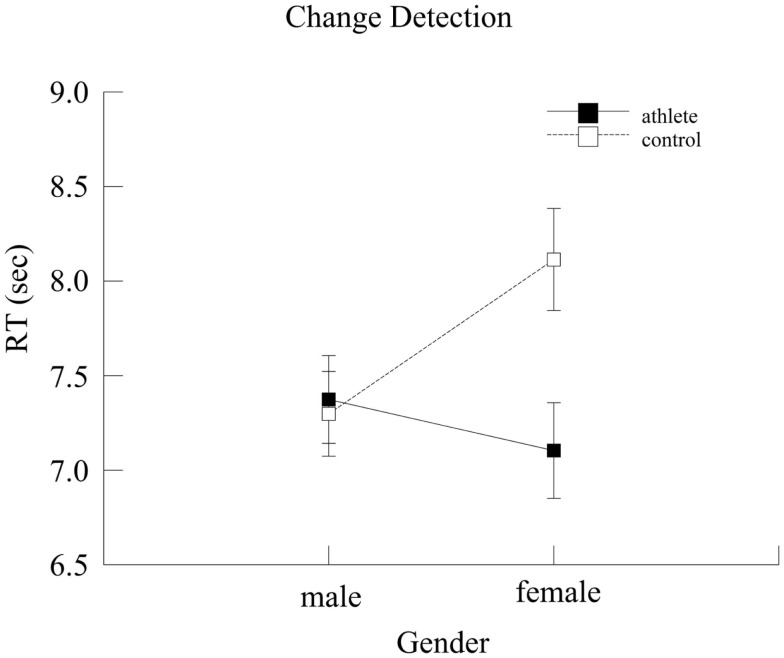
**Mean reaction time (s) for the two groups as a function of gender**. Error bars represent ±1 standard error.

Mean reaction time for correct trials was assessed through a univariate ANOVA. A marginal main effect of Group [*F*(1,136) = 3.65, *p* = 0.06, $\eta_{p}^{2}=0.03$] and a Group × Gender interaction [*F*(1,136) = 4.89, *p* = 0.03, $\eta_{p}^{2}=0.04$] were observed. Specifically, athletes (7.23 ± 0.17 ms) were faster than controls (7.70 ± 0.17 ms). A subsequent analysis of accuracy data indicated that this group difference was not due to a speed-accuracy tradeoff (*p* = 0.87). Gender differences were observed exclusively in the control group (*p* = 0.01), where men were significantly faster than women (7.29 ± 0.224 and 8.11 ± 0.27 ms, respectively). Male and female athletes did not differ (*p* = 0.36). In addition, *post hoc* analyses indicated that female athletes (7.10 ± 0.25 ms) were faster (*p* < 0.001) than female controls (8.11 ± 0.27 ms), and that this difference was not due to a possible speed-accuracy tradeoff. Specifically, female athletes were just as accurate as female controls (*p* = 0.39). Male athletes and male controls did not differ (*p* = 0.76). Results are displayed in Figure 6.

## Discussion

The present study examined whether expertise in sport is related to superior performance on measures of different aspects of perception and cognition. Specifically, we wanted to determine if elite volleyball players differed from non-athlete controls in tests of executive control, memory, and visuo-spatial attentional processing, as measured by the component skills approach. Our prediction that athletes would outperform non-athlete controls was based on the results of a recent meta-analysis by Voss et al. (2009), which showed that expertise in sport was related to high levels of performance on measures of processing speed and visual attention. In the attempt to fill specific gaps in the literature and overcome limitations of previous studies, the present study tested 87 elite athletes, employed a broad cognitive battery that included tasks that had not been previously used in the sport expertise literature, and examined perceptual-cognitive performance of male and female athletes belonging to two different age groups. Studies in the sport expertise literature usually report small, but positive effects that often lack statistical significance, which may be due to the small sample sizes of athlete groups. In the present study, despite the substantial sample size (compared to other studies), the effects obtained were mostly of small magnitude. Table 3 presents the main results of the study.

**Table 3:** **Summary of significant main effects and interactions**.

Tasks	Results		
	Group effect	Group × gender	Group × age
**TASK SWITCHING**			
Single trials	A faster than C		
**STOPPING**			
Go	C faster than A		
Stop	A faster than C		AC faster than JC
			JA faster than JC
Stop probability	A > C		JC > AC
			JA > JC
			AA > AC
**FLANKER**			
		MC faster than FC	
		FA faster than FC	
**CHANGE DETECTION**			
	A faster than C	MC faster than FC	
		FA faster than FC	

*A, athlete group; C, control group; FA, female athletes; MC, male controls; FC, female controls; AA, adult athletes; AC, adult controls; JA, junior athletes; JC, junior controls. Results refer to the RT measures of the cognitive constructs analyzed*.

Of primary interest to us were main effects of Group, indicating whether athletes differed from non-athlete controls (i.e., expertise effect), and the interactions of group with age, gender and important task-related factors. The results were partially in accordance with our hypotheses. The volleyball players differed from the non-athlete controls on three of the perceptual-cognitive tasks employed (two executive control tasks and one visuo-spatial attentional processing task). Overall, groups differed with respect to reaction time measures. Specifically, athletes were preferentially faster on the single trials of the Task Switching task, showed greater inhibitory control in the Stopping task, and were faster in detecting changes in the Change Detection task. *Post hoc* analyses indicated that these differences between athletes and controls were not due to a speed-accuracy tradeoff. In addition to these RT results, athletes showed a higher likelihood of stopping their prepotent response in the Stopping task (as reflected by the Stop Probability index).

These results support the prediction that transfer effects may be observed in those tasks that engage cognitive processes (and brain regions) analogous to the ones trained in volleyball. Executive functions are of fundamental importance to expert performance in volleyball. In addition, the Change Detection task has been shown to engage the prefrontal cortex, an area that is also involved in executive control (Beck et al., 2001).

On one task, the non-athlete group showed faster performance: controls were faster on Go trials (when no tone occurred) of the Stopping task and subsequent *post hoc* analyses showed that these differences were not due to a speed-accuracy tradeoff. This result favoring non-athletes is not in accordance with our predictions and will be discussed shortly.

Also of interest were Group × Gender interactions. We hypothesized that sport expertise may minimize gender differences. The predicted pattern was observed on two of the visuo-spatial attentional processing tasks: female and male athletes exhibited similar selective attention capacity (i.e., comparable speed in responding to relevant information while ignoring irrelevant information) in the Flanker task, and females were just as fast as males in detecting changes in visual scenes in the Change Detection task. On the other hand, male controls were faster than female controls on both tasks. Some of the differences in neuropsychological functioning of males and females have been attributed to culture and education (Caplan et al., 1997; Kimura, 1999), since training and practice appear to reduce gender differences in spatial ability (Chance and Goldstein, 1971; Connor et al., 1977). In this sense, it might be the case that gender differences within a sport on tasks involving perceptual-motor speed are minimized if male and female athletes are given equal opportunities for similar experiences, learning, and training (Ryan et al., 2004). This idea would explain why the gender differences, when they occurred, were only present in the control group in our study, not in the athlete group.

With respect to the Group × Age interactions, an interesting pattern of results was observed on the Stop RT and Stop Probability measures of the Stopping task: adult and junior athletes showed similar abilities in inhibitory control and precision in stopping their prepotent responses, while adult and junior controls were significantly different on both measures. Although teenagers usually perform worse than young adults in tasks where response time is a primary measure (Kail, 1986), the fact that junior athletes performed similarly to the adult athletes on a small subset of the tasks in the present study may be explained by a possible cognitive advantage (when compared to junior controls) provided by extensive sport training. It must be pointed out that age is confounded with years of experience in the present study. The adult athletes were, on average, 5 years older than the young athletes and had, on average, five more years of training. In this sense, it could be argued that the older athletes had “greater expertise” than the younger athletes. Although it is intuitive that expertise should increase with age, for the purposes of the present study we considered all athletes “experts,” despite the different amount of total sport training and the difference in age. The results obtained seem to indicate that this is indeed the case.

Two relevant factors need to be taken into account in the discussion of the small effect sizes obtained across the tasks of the cognitive battery. First and foremost, the emotional and physiological stress the adult athletes were under when they were tested might have negatively influenced their performance in the cognitive tasks. Emotional and physiological stress is known to significantly impact performance efficiency (Williams and Elliot, 1999; Williams et al., 2002). Although a certain level of stress is needed for optimal performance, the players who participated in the study had been in the training center for many weeks, training for major international competitions (including the Beijing Olympics). They were physically and emotionally stressed. In this context, a general effect of stress, across all measures of all tasks, could explain the small effect sizes obtained in the present study. A second factor, closely related to the first one, is motivation. Again, considering that our assessments were performed close in time to the World League and the Olympics, the athletes were focused on their preparation for competition.

Education is an additional factor. Because elite athletes in Brazil do not usually attend college, there was a significant difference in education between the adult players and their controls. Although education was not significant in the preliminary MANCOVAs, it would have been ideal if we could have selected athletes and controls with the same amount of formal education. Other factors, such as fatigue and time of day that the athletes were tested, could not be controlled. Some authors argue that testing athletes after training has a negative effect on test performance (Castiello and Umiltà, 1988), and most of the adult players were tested after training. However, analyses between athletes who were tested before training and those tested after training did not reveal any statistically significant differences between these groups in any of the tasks. Therefore, we can assume that “time of day” did not interfere in the athletes’ performance in the cognitive tests.

With respect to the unexpected superior performance of the control group on the Go condition of the Stopping task, a relevant issue needs to be pointed out. Although the Stopping task measures executive control, the Go condition does not (because participants are not required to inhibit their responses in this condition). Therefore, a fundamental difference observed in the Stopping task is that athletes showed superior performance on the two specific conditions that measure executive control, namely Stop RT and Stop Probability, while controls were faster on a less cognitively demanding RT measure. An alternative explanation is that these results reflect a specific strategy adopted by the athletes. The slower responses on the Go condition, combined with greater stop probabilities, could simply indicate that the athletes were less willing to make errors.

Thus, although our hypothesis that sport expertise might minimize gender differences was only supported by a limited number of measures and tasks in the present study, the expertise effects obtained substantiate the view that laboratory tests of cognition may indeed enlighten the sport-cognition relationship. The results suggest that the effects of sport expertise on perceptual and cognitive skills are reflected essentially in measures of response time, both in executive control and visuo-spatial attentional processing tasks, which is in accordance with the specific cognitive demands of volleyball. Sports characterized by performance under unpredictable conditions, especially fast ball games such as volleyball, require highly flexible attention (Anzeneder and Bosel, 1998). Evidence suggests that highly skilled volleyball players develop specific patterns of visual scanning (Ripoll, 1988) and, cognitively, may be quite flexible (Starkes and Alard, 1983). Finally, the results also suggest that women benefit to a greater extent from the cognitive advantage provided by sport expertise. Nevertheless, there is still much to be learned about cognitive-perceptual expertise in sports. A longitudinal study, tracking athletes along various levels, would be ideal to understand how cognitive abilities differ as a function of *a priori* broad cognitive abilities, experience (years of training), and type of training. Ultimately, the study of cognitive-perceptual expertise in sport has great potential to assist and guide trainers in the development of future expert athletes, and to provide insight into how brain structure and function differ following individual differences in sport experience.

## Conflict of Interest Statement

The authors declare that the research was conducted in the absence of any commercial or financial relationships that could be construed as a potential conflict of interest.
